# Spinally targeted paired associative stimulation with high-frequency peripheral component induces spinal level plasticity in healthy subjects

**DOI:** 10.1038/s41598-024-82271-4

**Published:** 2024-12-28

**Authors:** Anna Nätkynmäki, Leena Lauronen, Piia Haakana, Erika Kirveskari, Janne Avela, Anastasia Shulga

**Affiliations:** 1 BioMag Laboratory, HUS Diagnostic Center, Helsinki University Hospital, University of Helsinki and Aalto University School of Science, Helsinki, Finland; 2 Faculty of Sport and Health Sciences, University of Jyväskylä, Jyväskylä, Finland; 3 Clinical Neurophysiology, New Children’s Hospital, HUS Diagnostic Center, Helsinki University Hospital and University of Helsinki, Helsinki, Finland; 4 Motion Analysis Laboratory, New Children’s Hospital, Helsinki University Hospital and University of Helsinki, Helsinki, Finland; 5 Department of Physiology, University of Helsinki, Helsinki, Finland; 6 Clinical Neurophysiology, Clinical Neurosciences, HUS Diagnostic Center, Helsinki University Hospital and University of Helsinki, Helsinki, Finland; 7 NeuroMuscular Research Center, Faculty of Sport and Health Sciences, University of Jyväskylä, Jyväskylä, Finland; 8 Department of Physical and Rehabilitation Medicine, Helsinki University Hospital and University of Helsinki, Helsinki, Finland

**Keywords:** H-reflex, Interstimulus interval, Paired associative stimulation, Spinal plasticity, Transcranial magnetic stimulation, Spinal cord, Rehabilitation

## Abstract

A novel variant of paired-associative stimulation (PAS) consisting of high-frequency peripheral nerve stimulation (PNS) and high-intensity transcranial magnetic stimulation (TMS) above the motor cortex, called high-PAS, can lead to improved motor function in patients with incomplete spinal cord injury. In PAS, the interstimulus interval (ISI) between the PNS and TMS pulses plays a significant role in the location of the intended effect of the induced plastic changes. While conventional PAS protocols (single TMS pulse often applied with intensity close to resting motor threshold, and single PNS pulse) usually require precisely defined ISIs, high-PAS can induce plasticity at a wide range of ISIs and also in spite of small ISI errors, which is helpful in clinical settings where precise ISI determination can be challenging. However, this also makes the determination of high-PAS level of plasticity induction more challenging and calls for more research on the mechanism of action of high-PAS. We sought to determine if the TMS-induced orthodromic activation in upper motor neurons and PNS-induced antidromic activation in lower motor neurons arriving simultaneously to the intervening synapses at the spinal cord level can be shown to induce acute changes at the targeted location, unlike an otherwise identical but cortically targeted equivalent. Ten healthy subjects participated in two separate sessions, where high-PAS induced activation was set to target spinal (SPINAL) or cortical (CORTICAL) levels with ISI manipulation between otherwise identically applied TMS and PNS pulses. The outcomes were assessed with motor-evoked potentials (MEPs) and Hoffmann (H)-reflex before (PRE), immediately after, and 30 and 60 min after (POST, POST30, POST60) the intervention. MEPs were significantly enhanced in both interventions. In the SPINAL but not in the CORTICAL session, maximal H-reflex amplitudes significantly increased at two timepoints, indicating an increase in spinal excitability. The H/M ratio (maximal H-reflex normalized to maximal M-wave) also showed a significant increase from PRE to POST30 timepoint in the SPINAL session when compared with the CORTICAL equivalent. These results confirm that spinally targeted high-PAS with individualized ISIs indeed has an effect at the spinal level in the sensorimotor system. High-PAS is a novel PAS variant that has shown promising results in motor rehabilitation of individuals with SCI and these new findings contribute to the understanding of its mechanism of action. This provides further evidence for high-PAS as an option for clinical settings to target plasticity at different levels of the corticospinal tract.

**Supplementary Information:**

The online version contains supplementary material available at 10.1038/s41598-024-82271-4.

## Introduction

Spinal cord injury (SCI) can result in profound disability that reshapes multiple aspects of life^1^. After injury, both temporary and permanent alterations at the spinal cord require individuals to navigate changes that affect not only their physical and social lives but also their vocational roles, independence, and overall quality of life^1,2^. Recently, the prevalence of SCI completeness has changed more towards incomplete SCI^2,3^. Incomplete SCI means that some residual connections are preserved, which then opens possibilities in rehabilitation through plasticity induction to gain functionality and improve quality of life^1,4,5^.

Paired-associative stimulation (PAS) consists of transcranial magnetic stimulation (TMS) and peripheral nerve stimulation (PNS) and is one of the neuromodulation methods that has been studied when developing effective treatment for rehabilitation of people with SCI^4,5^. TMS has been widely adopted into clinical medicine over recent decades. For some patient populations, evidence-based guidelines exist for the use of repetitive TMS in rehabilitation^6,7^. PAS has not yet been transferred into routine clinical practice as part of SCI rehabilitation^4,5^. Promising results have already been obtained for individuals with incomplete SCI in improvement of motor performance and functional independence^5,8–16^. This study sought to address the need for more research and published data, as there is a need to increase in the number of patients that have undergone the procedure and to better understand mechanisms of action of PAS to further optimize the technique and to transfer it to clinical practice^17,18^.

PAS combines TMS and PNS to induce plastic changes in the desired location^19,20^. By synchronizing TMS applied at the primary motor cortex (M1) and PNS with a specific interstimulus interval (ISI), this non-invasive method can be used to elicit long-term potentiation (LTP)-like plasticity in a targeted location of the corticospinal sensorimotor system in healthy subjects^4,20^. By using precise ISIs, the activation induced by TMS and PNS pulses can be precisely targeted to synapses at cortical or spinal cord levels^13,19,21^. A modified variant of PAS, high-PAS, was designed with the specific aim to target and activate motor fibers in the spinal cord and therefore to induce plastic changes considered useful in SCI rehabilitation^4,22^. High-PAS utilizes individually targeted high-intensity TMS given with maximal stimulator output (MSO) and a high-frequency train of PNS to produce multiple activation volleys in the nervous system. High-PAS therefore differs from conventional PAS, which utilizes single-pulse TMS with intensity closer to resting motor threshold and single PNS pulses^4^. While conventional PAS requires precisely timed ISIs between two single pulses for simultaneous arrival at a target location, with high-PAS these induced multiple volleys produce several arrivals and create an LTP-like net effect and hence likely overcome possible long-term depression (LTD)-like changes^4,23^. Sjöström et al. (2001) explored the rate, timing, and number of afferents leading to LTP in a cell model^23^. Induced plasticity is usually thought to depend strongly on precise timing of firing and the number of cooperative inputs^23^. They found that instead of linear summation inside a close window of cooperative spike timing, conditions favour LTP and this wins over LTD^23^. Possible mechanisms of action for the high-PAS to cause alterations in the somatomotor system include synaptic reorganization, both spinally and supraspinally, rerouting interneurons, strengthening synaptic connections, and consequently improving conductivity^4,5^.

For ISI calculation, Shulga et al. (2021) have previously proposed the use of an F-response technique where F-response latency together with motor-evoked potential (MEP)-latency calculation is used to create a precise individual approximation for ISI in a PAS protocol^24^. F-responses are the late responses activated by PNS and reflect antidromic transmission from the stimulation site and through the corresponding motor neuron to the spinal cord and orthodromically back to the peripheral muscle^4,24^. Using the F-response technique in the calculation of ISI for lower-limb high-PAS, the timing of the arrival considers the cauda equina conduction times^4,24^.

In PAS, induced plasticity can be estimated with MEPs, which are TMS-evoked responses recorded from muscles with surface electromyography (EMG)^4,20^. This provides insights into the excitability of the corticospinal pathway by reflecting the responsiveness of motor cortex neurons and spinal motoneurons^25,26^. Even with small errors in temporal timing, high-PAS has been shown to double the MEP amplitudes measured in the lower limbs^4,17,27^. High-PAS with F-response technique for upper limbs is also possible^4,12^. In studies with individuals with chronic SCI that documented improvements in manual dexterity and movement functionality after multiple sessions of high-PAS, possible underlying plasticity mechanisms may include structural reorganization at the corticospinal-motoneuronal synapses of the spinal cord, altered conduction of neural fibers, and enhanced intraspinal interactions^8,11,12^. Even an acute session of paired TMS and PNS pulse stimulation targeting corticospinal-motoneuronal synapses can induce plastic changes in individuals with chronic SCI^14^. Changes were found in voluntary motor output measured with force and fine motor task and in MEP amplitudes measured with TMS, transcranial electrical stimulation, and cervicomedullary stimulation, suggesting modulation of residual synapses^14^. In healthy subjects after 50 pairs of conditioning paired TMS and PNS stimulation targeting corticospinal-motoneuronal synapses, increased cervicomedullary MEPs indicated altered interneuronal and synaptic transmission, suggesting spike-time-dependent plasticity at the spinal level in the corticospinal pathway^13^.

The changes at the spinal-cord level can also be investigated using the Hoffmann (H)-reflex^20,21^. The amplitudes of the H-reflex and the preceding M-wave, a direct motor response, initially grow along with increasing stimulus intensity according to Henneman’s Size Principle until maximal H-reflex (Hmax) is obtained^28^. Hmax thus reflects the strongest activation of the reflex arc. H-reflex diminishes with further increasing stimulus intensity and diminishes away with supramaximal stimulation intensity where M-wave reaches its maximal amplitude (Mmax). Mmax reflects the maximal muscle activation in which all motor units available have been recruited^28–30^. In Leukel et al. (2012), the observed adaptations at the spinal level produced by combining 360 pulses of cortical TMS and electrical stimulation from the tibial nerve timed to arrive at the spinal level were measured with cervicomedullary TMS-conditioned H-reflexes. TMS was delivered with threshold intensity and electrical stimulation with intensity able to elicit H-reflexes with a size of 15–25% of the maximum M-wave^21^. In Cortez et al. (2011), a single 15-min session of repetitive electrical stimulation from the periphery with intensity able to elicit conditioned H-reflex of 0.5–1 mV peak-to-peak amplitude and subthreshold transcranial stimulation facilitated H-reflex amplitude. However, apart from clinical functional and motor improvements documented in patients with incomplete SCI, evidence for spinal level action has not been studied for spinally targeted high-PAS.

PAS was targeted at the cortical level in early studies^19,20^. However, as previously described, PAS can also be targeted to the spinal-cord level and can improve recovery after SCI^5,13^. We sought to determine by using H-reflex measurements if spinally targeted high-PAS can induce acute changes at the spinal level, and if these changes are more pronounced than those possibly induced by otherwise identical but cortically targeted high-PAS^4^. We conducted two experimental sessions where healthy subjects received in randomized order one high-PAS session targeted to the spinal-cord level and another session targeted to the cortical level. We hypothesized that both experiments would show increased corticospinal excitability observed by MEP enhancement, but spinally targeted high-PAS would show a more significant increase in Hmax amplitudes than cortically targeted high-PAS, thus representing an enhanced modification in spinal excitability by spinally targeted high-PAS.

## Methods

### Subjects

Ten healthy volunteers (8 females, 2 males; age 21–42 years, mean 28 years; 9 right, 1 left lower-limb dominant) participated in the study. Leg dominance was assessed by asking which leg they prefer in a football penalty kick. All subjects provided signed written consent to participate in the study and to undergo magnetic resonance imaging (MRI). MRI was conducted with a Siemens Magnetom Skyra 3 Tesla machine providing high-resolution 3D images. With axial and coronal reconstruction, T1 Magnetization Prepared Rapid Gradient Echo (MPR) in sagittal plane and T2 Sampling Perfection with Application optimized Contrasts (SPC) in the sagittal plane, 3D images were obtained with 0-mm slice gap, 1-mm slice thickness, and 256 × 256 pixel matrix. Subjects were asked to follow their normal daily routines and to not engage in new or abnormally burdensome physical activity 24 h prior to the high-PAS session. The subjects did not have any contraindications to TMS, such as magnetic or metal objects or medical devices. Exclusion criteria for participation included cardiac or neurological diseases, drug or alcohol abuse, pregnancy, or neurologically active medication. Subjects were reminded of their right to withdraw from the study at will at any time. All safety, ethical, and application guidelines were followed as closely as possible^31^.

### Experimental design

**Fig. 1:**
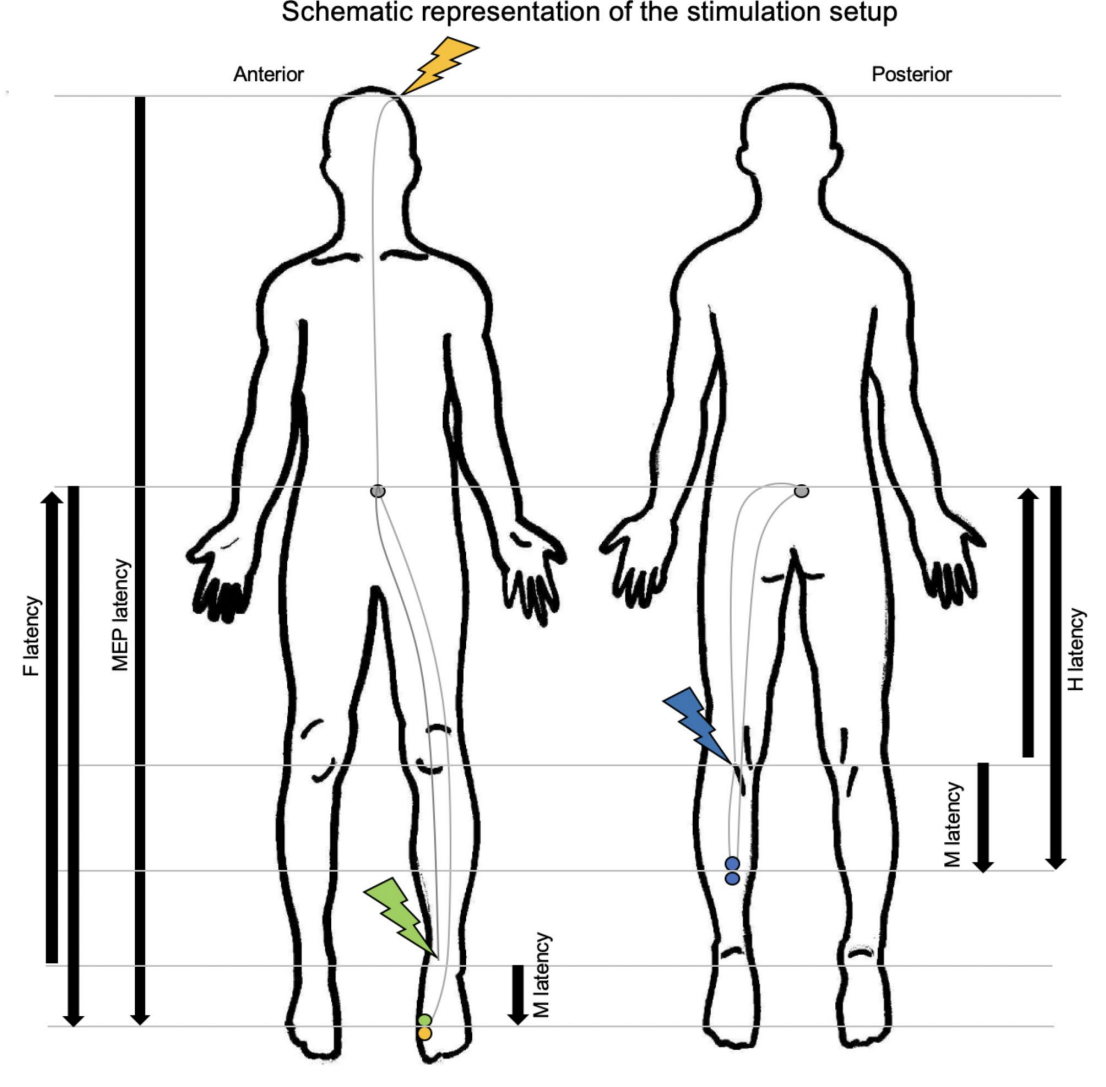
Schematic representation of the stimulation setup. TMS was applied above the M1 hotspot (orange lightning) to target cortical representation of the abductor hallucis muscle. For F-response and PNS, the tibial nerve was stimulated behind the medial malleolus (green lightning) with bipolar electrode placement along the nerve trunk. Responses were recorded over the abductor hallucis muscle (green and orange dots). In H-reflex measurements, the tibial nerve was stimulated from the popliteal fossa (blue lightning), with manually applied bipolar electrode placement horizontally over the nerve trunk and the responses recorded over the soleus muscle (blue dots). Spinally targeted ISI was calculated as ${F}_{latency}- {MEP}_{latency}$. ${MEP}_{latency}$ was used for cortically targeted ISI.

Two high-PAS sessions were scheduled in a randomized order. In one session, 20-min high-PAS was targeted such that the arrival of the TMS and PNS pulses was estimated to occur spinally (SPINAL) and another cortically (CORTICAL). A single high-intensity TMS pulse was timed with predetermined ISI to coincide with the first pulse of the PNS train applied from the peripheral nerve trunk via electrical stimulation such that the arriving pulses coincide at the desired level, either spinally or cortically. High-PAS ISIs were adjusted individually for every subject and session. To precisely target the pulses arrival to the spinal-cord level, the required ISI was computed using the formula ${F}_{latency}-{MEP}_{latency}$, as described by Shulga et al. (2021) (Fig. 1). To target the cortical level, ${MEP}_{latency}$ was used as ISI. All high-PAS sessions were applied with a minimum of 1 week between the sessions to avoid any possible carryover effects from the previous session^32^. Subjects were blinded to session order.

**Fig. 2:**
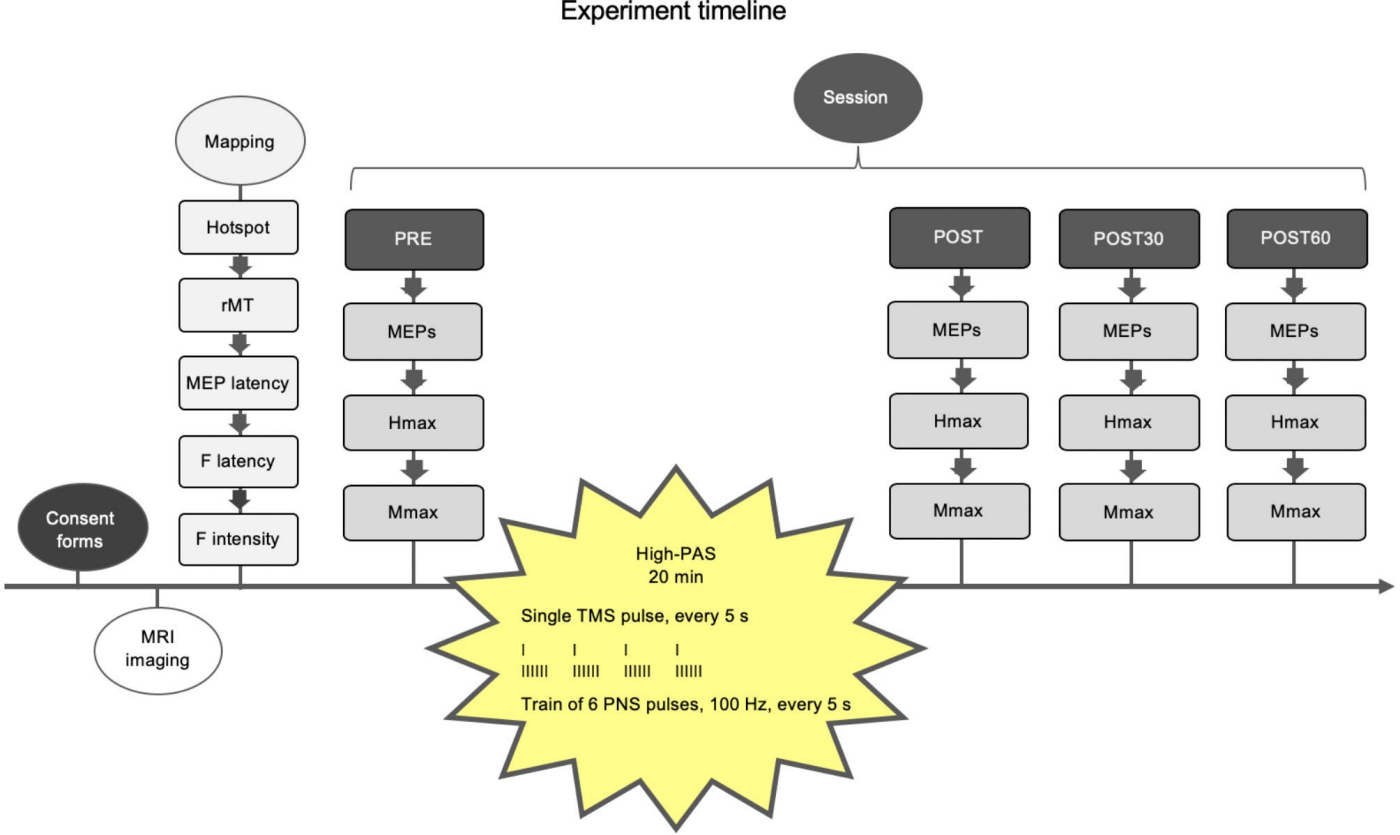
Experiment timeline. All subjects provided signed written consent (consent forms) and underwent MRI. There was a separate day for mapping (mapping) prior to the experimental sessions (session). All measurement variables (MEPs, Hmax, Mmax) were measured before (PRE), immediately after, and 30 and 60 min after (POST, POST30, POST60) the high-PAS intervention. The only difference between experimental sessions was ISI between TMS and PNS pulses in the spinally and cortically targeted high-PAS intervention.

The experiments followed an identical structure and protocols (Fig. 2) with the only difference being the applied ISI during 20-min high-PAS. Prior to the sessions, a separate day was allocated for a mapping session. This included determining the hotspot and resting motor threshold (rMT), MEP latency, F-response latency, and stimulation intensity (see “Transcranial magnetic stimulation” and “F-response measurement and peripheral nerve stimulation”). The outcomes of the experiments were assessed with TMS-induced MEPs and PNS-induced Hmax before (PRE), immediately after, and 30 and 60 min after (POST, POST30, POST60) the intervention.

### Transcranial magnetic stimulation

TMS was administered using a cooled figure-of-eight coil (outer diameter 70 mm, eXimia magnetic stimulator, Nexstim Ltd., Helsinki, Finland). 3D MRIs were downloaded to the Nexstim device, which reconstructed 3D images allowing navigation. Navigated TMS (nTMS) system featured a head tracker, strategically placed on the subject’s forehead just above the eyebrows, which allowed systematic placement and therefore reducing errors in calibration. Through this MRI-guided navigation, constructed 3D MRI models unique to each subject allowed coil localization and induced electric field in M1 with high accuracy and reproducibility^33^. Using navigation, the same cortical location was targeted with precise coil orientation and inclination securing the exact magnetic field throughout the experiments^27^.

TMS was targeted to activate the abductor hallucis (AH) muscle from the non-dominant lower limb. This muscle has been commonly chosen in our previous healthy-subject experiments because of small intrinsic variability in MEP responses (for example^9,24,27,32,34^). AH is also often a muscle that no subject is highly skilled in the use of, which creates a more neutral and controlled environment. For all subjects except one, the coil was positioned over the right hemisphere M1 to excite the optimal stimulation site for AH (the hotspot). The coil was placed over the left hemisphere M1 for the one subject with left leg dominance. Mapping sessions included mapping the hotspot, resting motor threshold (rMT), and MEP latency in respective order (Fig. 2) (see Shulga et al. 2021 for a more detailed procedure)^4^. At the outset, induced current was perpendicularly oriented to the sulcus. With an integrated EMG device, eXimia stimulator was used to record and analyse MEPs (band-pass filter 10–500 Hz, sampling rate 3 kHz). EMG electrodes (Neuroline 720, AMBU A/S, Ballerup, Denmark) were placed over the belly of the AH muscle and over the distal side of the first metatarsophalangeal joint. A ground electrode was placed over the bony surface of the stimulated foot’s bridge. rMT was determined by inducing at least 5/10 MEPs with peak-to-peak amplitude > 50 µV with the lowest TMS intensity possible over the hotspot of the AH muscle. Prior to the experiments,15 MEPs were recorded with 3.3-s intervals with an intensity set at 120% rMT to obtain an averaged value of MEP latency required to calculate ISIs for the high-PAS sessions. During the experiments, 30 MEPs were averaged in amplitude to present excitability individual to timepoint. Recordings were conducted at resting state. The background EMG level was visually observed online and baseline activity did not exceed 50 µV in any subject. Any MEPs with spontaneous activation detected during 200 ms prestimulus time were excluded from the analysis.

### F-response measurement and peripheral nerve stimulation

The mapping session included determination of F-response latency for ISI calculation and PNS intensity for high-PAS intervention for every subject, as previously described^4,24,32^. F-responses were recorded and analysed online with a Dantec Keypoint^®^ electroneuromyography device (Natus Medical Inc., California, USA). The stimulating electrodes were placed between the Achilles tendon and medial malleolus to reach the tibial nerve. The arrangement of recording electrodes for the AH muscle was consistent with that employed during the TMS measurements. The F-response latency required for ISI calculation was assessed with a single 0.2-ms stimulation pulse with suprathreshold intensity^24,32^. In online analysis, the shortest latency of a signal deviating from the baseline out of 10 F-responses was chosen. The PNS intensity used in high-PAS intervention was assessed with a single 1-ms pulse and determined to be the minimum intensity that evoked a reliable F-response in at least 1/10 pulses^24,32^.

### Paired-associative stimulation

High-PAS was constructed by combining TMS and PNS at a frequency of 0.2 Hz such that pulses arrived in every 5 s triggered by Presentation^®^ software (Neurobehavioral Systems Inc., California, USA). The high-PAS session took 20 min. A single-pulse TMS was applied with 100% MSO and PNS with 100 Hz train of 6 biphasic pulses with pulse width 1 ms, creating the high-PAS protocol^4^. The TMS-elicited volley was aimed to arrive simultaneously with the first volley of the PNS train at the level targeted. To reduce the unpleasant sensation of the electric pulses, Emla (2.5% lidocaine/prilocain cream), which penetrates approximately 3–5 mm into the skin, was applied over the stimulated area approximately 30 min before high-PAS^4,35^. High-PAS was administered to the subjects when they were in a relaxed state. If necessary, the subjects received guidance to adjust their sitting position for optimal comfort and relaxation. Additionally, continuous monitoring of the coil position was maintained.

### H-reflex measurements

H-reflexes were stimulated, recorded, and analysed online with a Dantec Keypoint.net^®^ electroneuromyography device (Natus Medical Inc., California, USA) while subjects laid prone^29,36^. The EMG signal was recorded at a sampling frequency of 48 kHz and filtered between 20 Hz and 10 kHz. When positioning the subjects, we sought to create as relaxed a state as possible for the subjects with particular interest on repeatability of the position. Recording electrodes were positioned on the soleus muscle 2–3 cm apart and ground electrode on bony surface of the lateral or medial side of the knee. With bipolar (diameter 7 mm, interelectrode distance 1.2 mm from the center, Natus Medical Inc., California, USA) stimulating electrode placement, monophasic 0.5 ms electrical pulses were delivered at the popliteal fossa horizontally over the tibial nerve trunk. Individual stimuli were applied manually with various time intervals lasting several seconds to avoid excessively frequent stimulation that could cause post-activation depression and alter the reflex measurements^28^. The intensity of stimuli was increased with individual steps to acquire single Hmax amplitude. Intensity was fluctuated to ensure real maximal value around the suspected single maximal amplitude. After obtaining a representative Hmax amplitude of a current condition and increasing the intensity, H-reflex disappeared and with supramaximal intensity, Mmax amplitude reached its stable maximum value. Amplitudes and latencies of variables were analysed online and verified offline.

### Statistical analysis

All data were analysed with IBM SPSS Statistics software (version 29). Shapiro-Wilk test was used to test data normality. Non-parametric tests were selected, as several variables were not normally distributed and given the small sample size. MEP and reflex data were analysed separately, excluding correlation analysis. Percentage changes within session for multiple comparisons indicating differences across timepoints were analysed using Friedman’s two-way ANOVA for related samples defined with the Dunn-Bonferroni test. If Friedman’s test showed significance, the later timepoints were individually compared to the PRE timepoint with post-hoc pairwise comparison. Therefore, the Dunn-Bonferroni correction was set to 3. Wilcoxon 2-related samples, with statistical significance level *p* < 0.05, was used to test between-group differences in accordance to timepoint. All percentage changes used in statistical analyses were calculated individually as $\frac{POST\, (x)}{PRE}*100$. In figures presented, percentage changes are calculated as $\frac{POST\, (x)}{PRE}*100-100$, which indicates the amount in percentage the value increased or decreased relative to PRE value. In correlation analyses, Pearson correlation coefficient with significance level set to *p* < 0.05 was used with 95% confidence interval.

### Ethics statement

The study was approved and reviewed by the Helsinki University Hospital Regional Committee on Medical Ethics in accordance with the declaration of Helsinki. Every subject provided signed written informed consent for participation and for undergoing MRI. Subjects were fully informed of the procedures and the risks involved in the study. Every subject was informed of their right to withdraw from the study at will at any time.

## Results

**Fig. 3:**
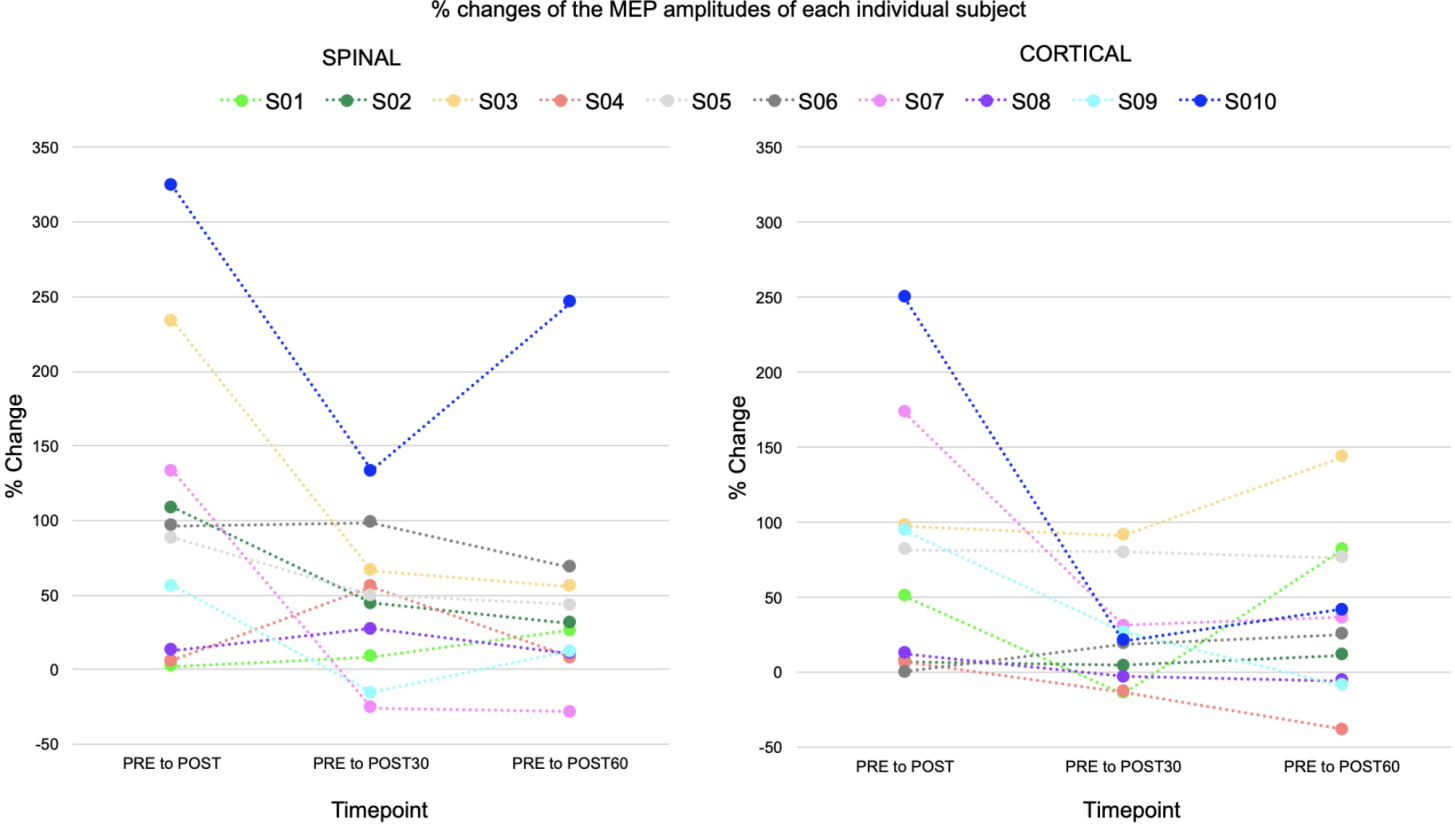
Percentage changes of the MEP amplitudes of each individual subject (*n* = 10). The colours represent the same subjects. Changes from PRE to POST, POST30, and POST60 timepoints in the SPINAL and the CORTICAL session.

**Fig. 4:**
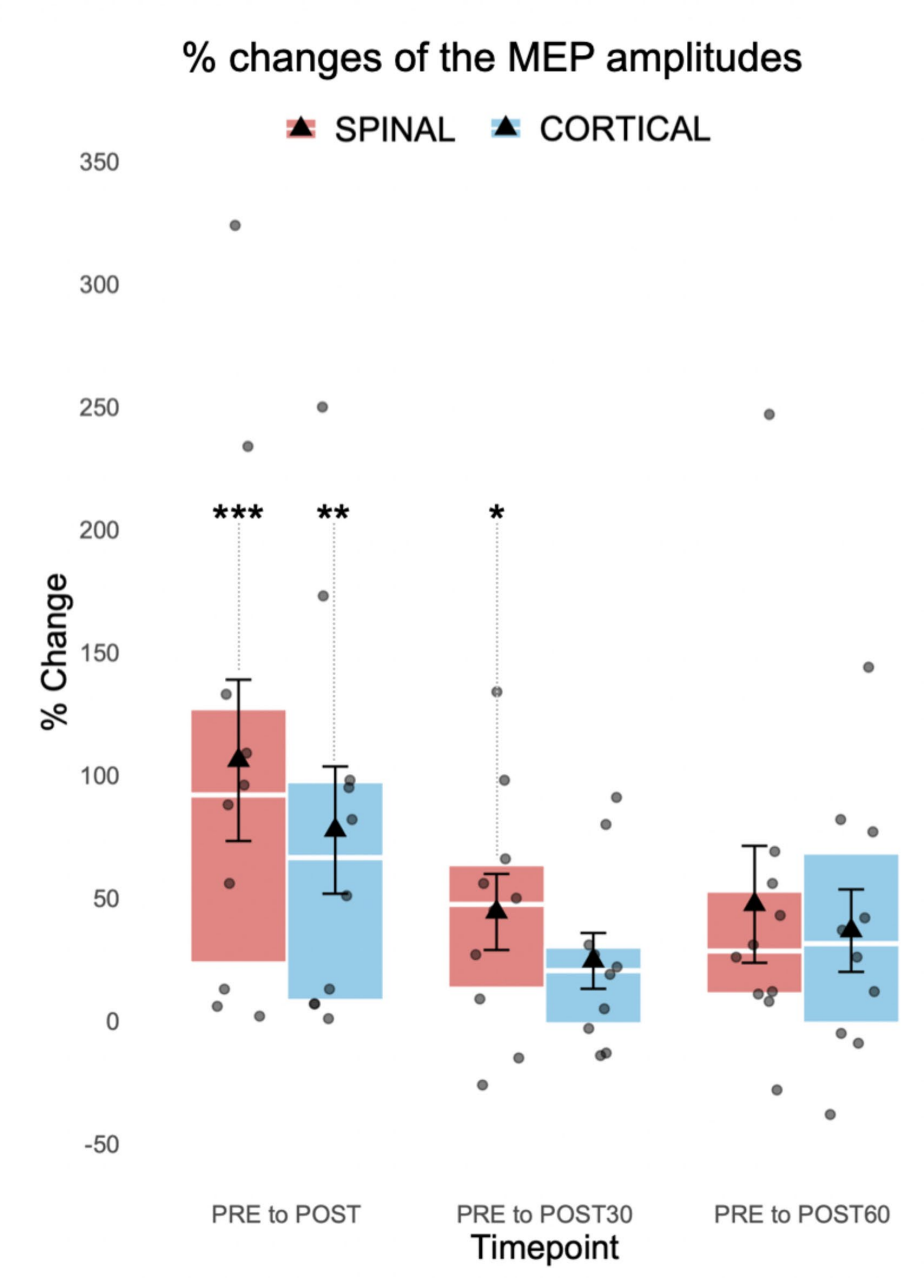
Percentage changes of the MEP amplitudes (*n* = 10). Changes from SPINAL PRE timepoint to POST (*p* < 0.001***), POST30 (*p* = 0.017*), and POST60 (*p* = 0.170) timepoints and from CORTICAL PRE timepoint to POST (*p* = 0.003**), POST30 (*p* = 0.896), and POST60 (*p* = 0.170) timepoints. Each box represents the interquartile range of the data with error bars (whiskers), mean (triangle), median (line), individual subjects (dots), and statistical significance (***p < 0.001, **p < 0.01, *p < 0.05).

Percentage changes of the MEP amplitudes of each individual subject are presented in Fig. 3. The MEPs were significantly enhanced from PRE to POST timepoint in both the SPINAL (*p* < 0.001, mean increase 352 µV) and the CORTICAL (*p* = 0.003, mean increase 309 µV) sessions (Fig. 4). A significant increase was also observed from PRE to POST30 timepoint (*p* = 0.017, mean increase 151 µV) in the SPINAL session. The CORTICAL POST30 (*p* = 0.896, mean increase 80 µV) did not show significant increase. Also, increases to POST60 did not show a significance (SPINAL *p* = 0.170, mean increase 143 µV; CORTICAL *p* = 0.170, mean increase 100 µV). No significant differences were observed when MEP amplitude changes from the PRE timepoint to individual later timepoints were compared between sessions (POST *p* = 0.241; POST30 *p* = 0.386; POST60 *p* = 0.959).

**Fig. 5:**
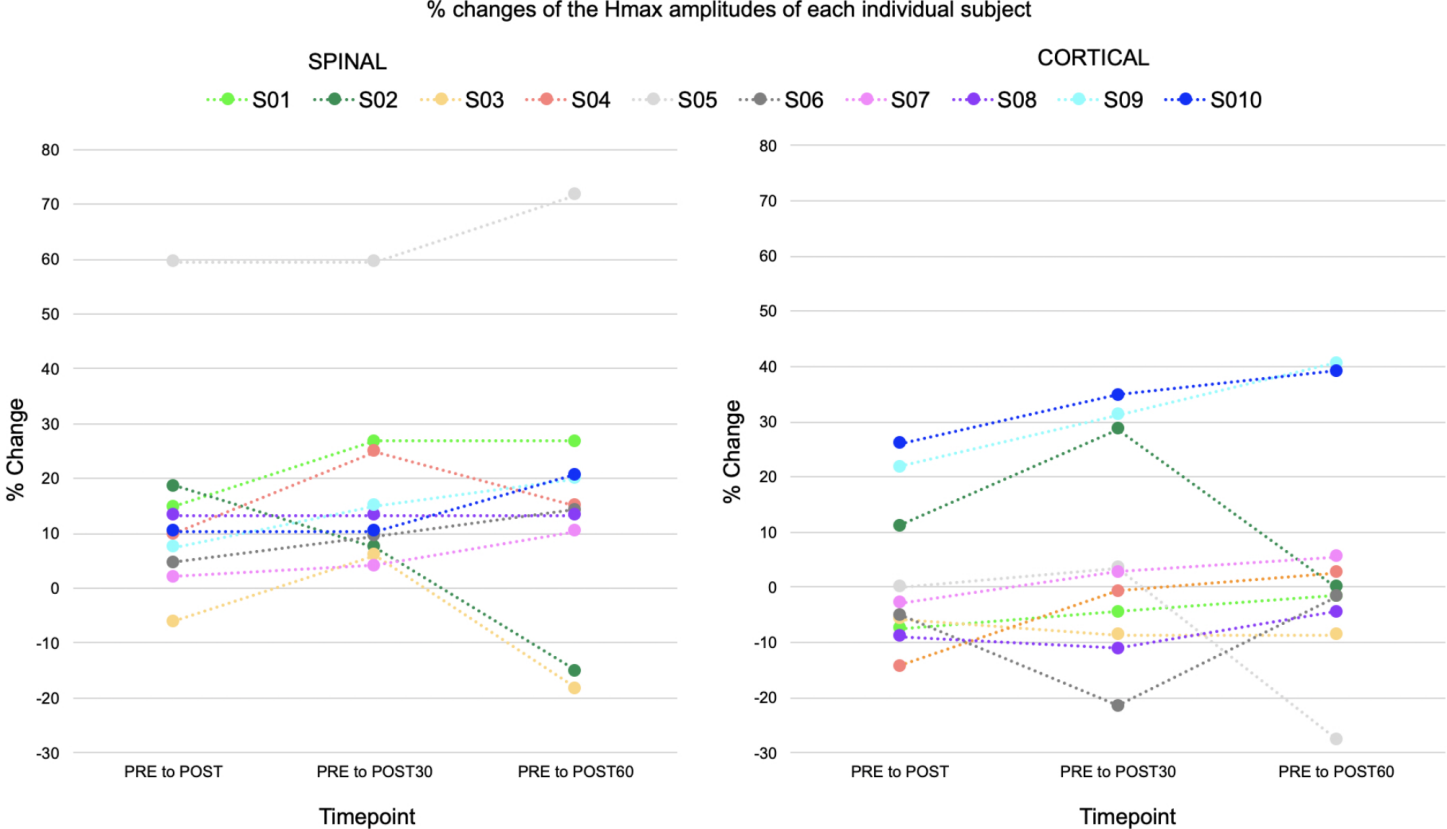
Percentage changes of the Hmax amplitudes of each individual subject (*n* = 10). The colours represent the same subjects. from PRE to POST, POST30, and POST60 timepoints in the SPINAL and the CORTICAL session.

**Fig. 6:**
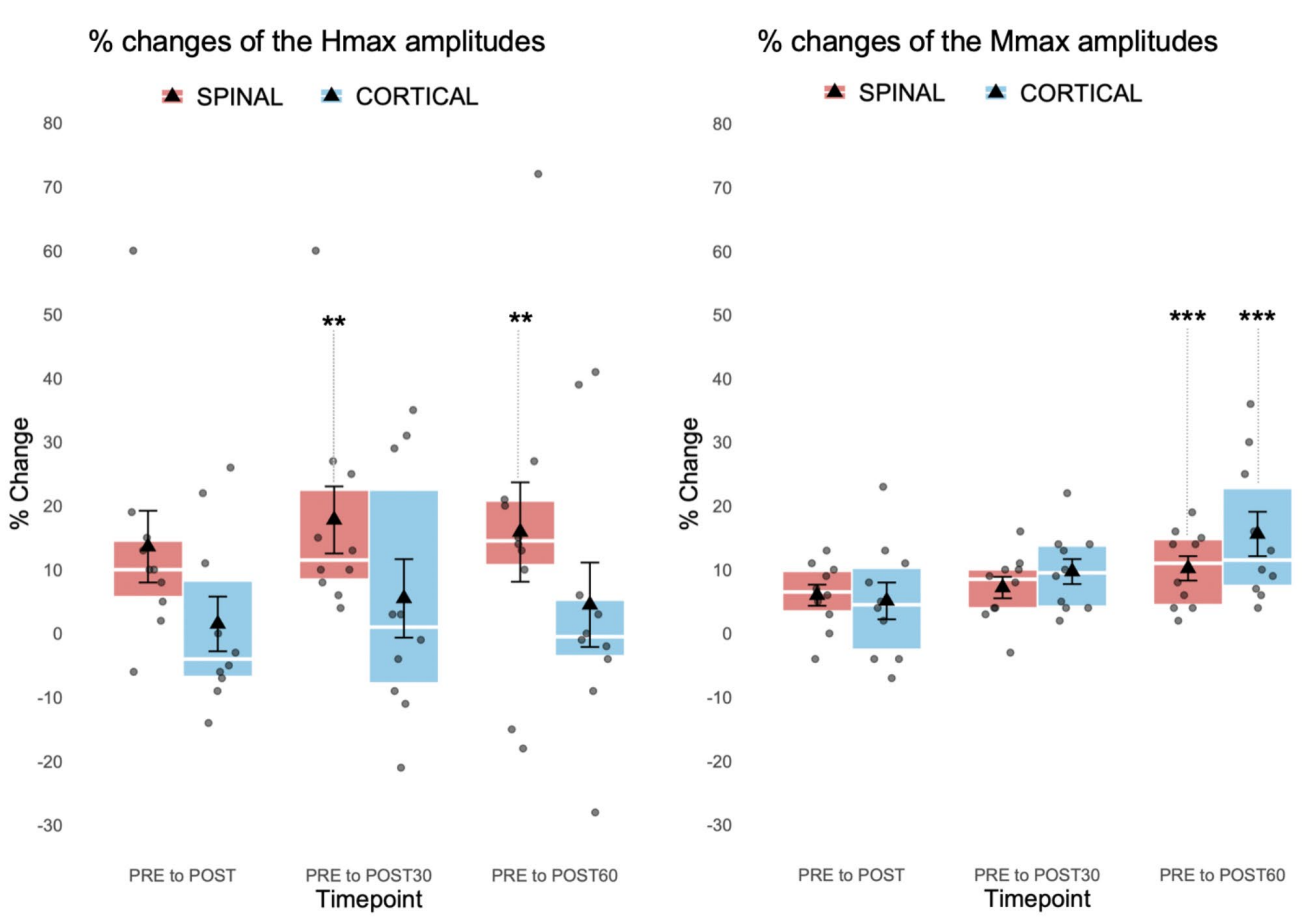
Percentage changes of the Hmax and Mmax amplitudes (*n* = 10). Changes from SPINAL PRE timepoint to POST (Hmax *p* = 0.170, Mmax *p* = 0.058), POST30 (Hmax *p* = 0.004**, Mmax *p* = 0.073), and POST60 (Hmax *p* = 0.004**, Mmax *p* < 0.001***) timepoints and from CORTICAL PRE timepoint to POST (Hmax *p* = 0.582, Mmax *p* = 0.300), POST30 (Hmax *p* = 1.995, Mmax *p* = 0.073), and POST60 (Hmax *p* = 2.187, Mmax *p* < 0.001***) timepoints. Each box represents the interquartile range of the data with error bars (whiskers), mean (triangle), median (line), individual subjects (dots), and statistical significance (***p < 0.001, **p < 0.01).

Percentage changes of the Hmax amplitudes of each individual subject are presented in Fig. 5. After high-PAS in the SPINAL session, mean Hmax amplitudes increased significantly from PRE to POST30 (*p* = 0.004, mean increase 0.543 mV) and to POST60 (*p* = 0.004, mean increase 0.535 mV) timepoints (Fig. 6), whereas no significant change was observed in PRE to POST (*p* = 0.170, mean increase 0.352 mV). In contrast to the SPINAL session, no significant changes in Hmax amplitudes were observed in the CORTICAL session (*p* = 0.373) at any timepoints (POST mean decrease 0.014 mV; POST30 mean increase 0.068 mV; POST60 mean increase 0.101 mV). At the individual subject level, in the SPINAL session, Hmax amplitudes showed increases in 8/10 subjects across all timepoints. In the CORTICAL session, a similar trend was observed in only 2/10 subjects. When Hmax amplitude changes from PRE timepoint to later timepoints were compared between sessions, no significant differences were observed (POST *p* = 0.114; POST30 *p* = 0.114; POST60 *p* = 0.508).

In addition, increases were observed in Mmax amplitudes in both the SPINAL and the CORTICAL session (Fig. 6). The mean change from PRE to POST60 was significant in both sessions (SPINAL *p* < 0.001, mean increase 0.55 mV; CORTICAL *p* < 0.001, mean increase 0.63 mV). However, the increases from PRE to POST (SPINAL *p* = 0.058, mean increase 0.35 mV; CORTICAL *p* = 0.300, mean increase 0.23 mV) and POST30 (SPINAL *p* = 0.073, mean increase 0.40 mV; CORTICAL *p* = 0.073, mean increase 0.42 mV) were not significant. When the Mmax amplitude changes from PRE timepoint to later timepoints were compared between sessions, no significant differences were observed (POST *p* = 0.386; POST30 *p* = 0.445; POST60 *p* = 0.203).

**Fig. 7:**
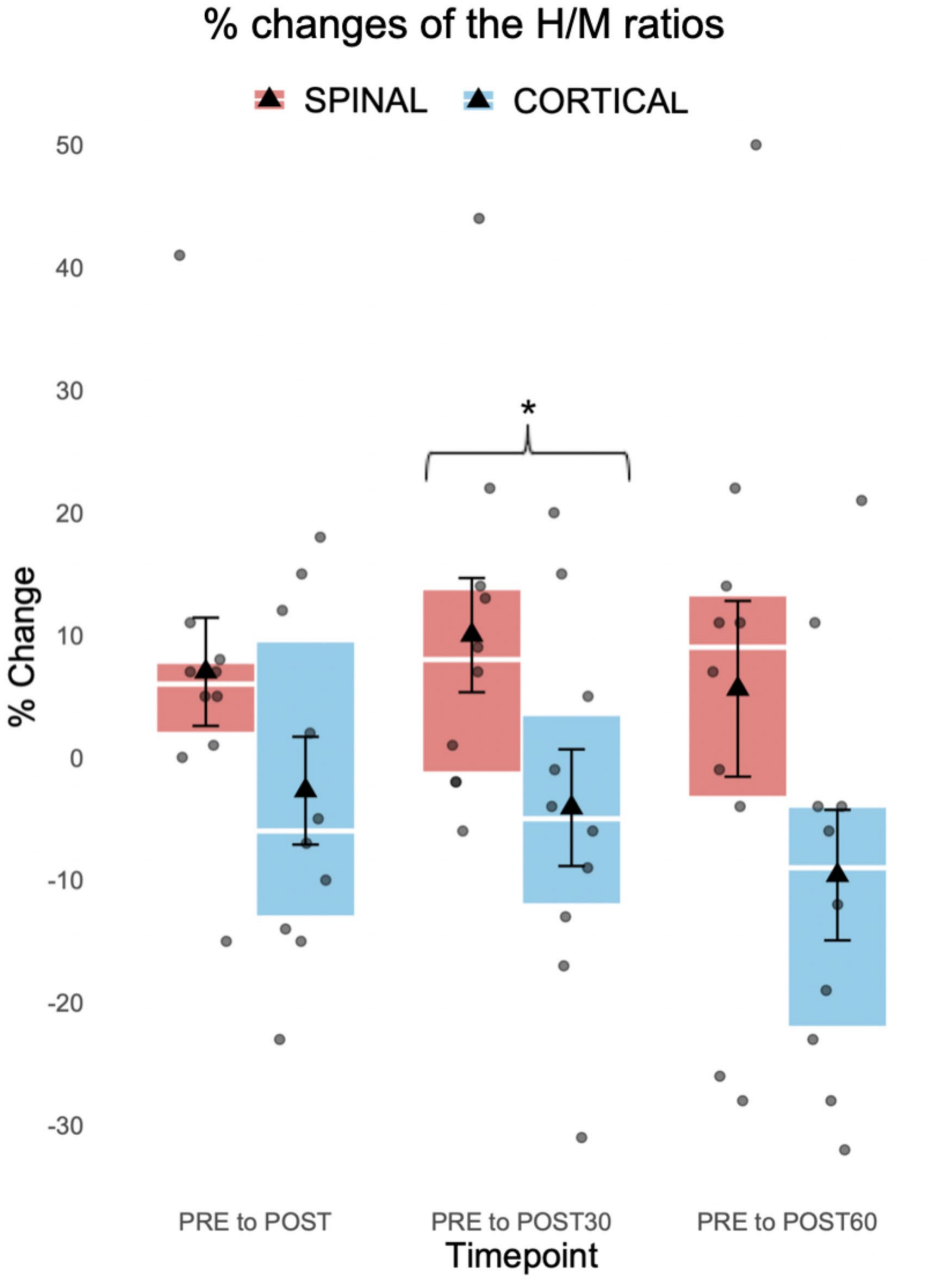
Percentage changes of the H/M ratios (*n* = 10). Significances when comparing sessions within timepoint from PRE timepoint to POST (*p* = 0.093), POST30 (*p* = 0.037*), and POST60 (*p* = 0.074) timepoints in the SPINAL and the CORTICAL session. Each box represents the interquartile range of the data with error bars (whiskers), mean (triangle), median (line), individual subjects (dots) and statistical significance (*p < 0.05).

Each Hmax amplitude was normalized to the corresponding Mmax amplitude creating a H/M ratio. Although there were no significant increases in H/M ratios within sessions (SPINAL *p* = 0.265; CORTICAL *p* = 0.218), one significant difference was observed between sessions (Fig. 7). The mean increase of 0.054 from PRE to POST30 timepoint in the SPINAL session was significantly larger (*p* = 0.037) than the observed mean decrease by 0.033 from PRE to POST30 in the CORTICAL session. Similar trends were also observed between changes in PRE to POST (*p* = 0.093, SPINAL mean increase by 0.027 and CORTICAL mean decrease by 0.029), and POST60 timepoints (*p* = 0.074, SPINAL mean increase 0.037 and CORTICAL mean decrease by 0.052).

No statistically significant correlations were identified between the changes in MEP and Hmax or Mmax amplitudes or in the H/M ratios. The changes in Hmax and Mmax amplitudes also did not correlate with each other. Although changes in Mmax amplitudes did not show significant correlations with H/M ratios, changes in Hmax amplitudes did, indicating inherent influence of Hmax in the H/M ratio, unlike Mmax. All correlation results are shown in Table 1.

**Table 1:** Correlations between changes (%) in the MEP and Hmax amplitudes, MEP and Mmax amplitudes, MEP amplitudes and H/M ratios, Hmax and Mmax amplitudes, hmax amplitudes and H/M ratios, and Mmax amplitudes and H/M ratios analysed across timepoints PRE to POST, PRE to POST30, and PRE to POST60 for both SPINAL and CORTICAL sessions (*n* = 10).

Variables	Session	PRE to POST			PRE to POST30			PRE to POST60		
		r	Sig.	95% CI	r	Sig.	95% CI	r	Sig.	95% CI
MEP, Hmax	SPINAL	−0.225	0.532	B [−0.972, 0.265] F [−0.749, 0.472]	−0.025	0.946	B [−0.571, 0.493] F [−0.644, 0.614]	0.065	0.857	B [−0.562, 0.563] F [−0.588, 0.668]
	CORTICAL	0.586	0.075	B [−0.154, 0.925] F [−0.069, 0.888]	−0.054	0.883	B [−0.491, 0.545] F [−0.661, 0.596]	−0.374	0.288	B [−0.776, 0.137] F [−0.812, 0.335]
MEP, Mmax	SPINAL	−0.374	0.288	B [−0.765, 0.336] F [−0.751, 0.468]	0.063	0.862	B [−0.680, 0.940] F [−0.590, 0.666]	0.004	0.991	B [−0.528, 0.900] F [−0.627, 0.632]
	CORTICAL	0.111	0.759	B [−0.630, 0.842] F [−0.557, 0.692]	0.041	0.910	B [−0.628, 0.751] F [−0.604, 0.654]	−1.590	0.660	B [−0.634, 0.338] F [−0.717, 0.523]
MEP, H/M ratio	SPINAL	−0.170	0.638	B [−0.741, 0.535] F [−0.722, 0.515]	−0.033	0.927	B [−0.654, 0.509] F [−0.649, 0,609]	0.065	0.858	B [−0.635, 0.448] F [−0.589, 0.667]
	CORTICAL	0.434	0.210	B [−0.266, 0.906] F [−0.269, 0.835]	−0.069	0.849	B [−0.533, 0.483] F [−0.670, 0.586]	−0.339	0.337	B [−0.701, 0.170] F [−0.798, 0.369]
Hmax, Mmax	SPINAL	0.491	0.150	B [−0.347, 0,881] F [−0.201, 0.856]	0.233	0.518	B [−0.362, 0.727] F [−0.465, 0.752]	−0.046	0.899	B [−0.726, 0.523] F [−0.657, 0.601]
	CORTICAL	0.141	0.698	B [−0.490, 0.697] F [−0.536, 0.708]	0.561	0.092	B [−0.217, 0.937] F [−0.106, 0.880]	0.300	0.400	B [−0.174, 0.844] F [−0.406, 0.782]
Hmax, H/M ratio	SPINAL	0.948**	< 0.001	B [0.260,0.992] F [0.791,0.988]	0.937**	< 0.001	B [0.608, 0.988] F [0.749, 0.985]	0.963**	< 0.001	B [0.849, 0.992] F [0.847, 0.991]
	CORTICAL	0.829**	0.003	B [0.556, 0.946] F [0.416, 0.958]	0.955**	< 0.001	B [0.897, 0.994] F [0.815, 0.990]	0.902**	< 0.001	B [0.445, 0.986] F [0.631, 0.977]
Mmax, H/M ratio	SPINAL	0.189	0.600	B [−0.698, 0.780] F [−0.500, 0.732]	−0.121	0.739	B [−0.829, 0.595] F [−0.698, 0.550]	−0.313	0.379	B [−0.885, 0.369] F [−0.787, 0.394]
	CORTICAL	−0.433	0.212	B [−0.864, 0.308] F [−0.835, 0.271]	0.290	0.416	B [−0.620, 0.843] F [−0.416, 0.778]	−0.136	0.708	B [−0.824, 0.742] F [−0.705, 0.540]

r, Pearson correlation coefficient; sig., level of significance; B [lower, upper] 95% confidence interval using the bootstrap method based on 1000 bootstrap samples; F [lower, upper], Fisher’s r-to-z transformation with bias adjustment. **Correlation is significant at the 0.01 level (2-tailed).

## Discussion

This study revealed the effects of spinally and cortically targeted high-PAS in healthy subjects. As anticipated, both interventions demonstrated high-PAS-induced potentiation of MEP amplitudes indicating enhanced corticospinal excitability. The high-PAS intervention in the SPINAL session induced enhanced Hmax amplitudes across all timepoints, with statistical significance observed at two timepoints. In addition, the H/M ratio increased from the PRE to POST30 timepoint and the increase was significantly larger in the SPINAL session compared to the CORTICAL session. When focusing on the Hmax amplitude results of individual subjects, the observed trend supports the changes detected at the spinal-cord level. These results demonstrate that the presented ISI calculation enables timing of the pulses’ arrival and succeeded activation targeted at the spinal-cord level during high-PAS stimulation. This spinally targeted high-PAS led to alterations in the corticospinal and spinal-level excitability, unlike high-PAS that targets the cortical level, which did not lead to similar spinal-level changes. These results on overall excitability changes may reflect the phenomena underlying the improvement seen in our previous clinical studies of patients with chronic motor-incomplete SCI receiving a spinally targeted high-PAS protocol^8,10,12,17^ (see Shulga et al. 2021). Our previous studies have included patients with paraplegia and tetraplegia and have shown regained ankle functions, grasping, standing, and supported walking and improved functionality in daily life^8,10,17^. These clinical findings show that at least in some patient groups, high-PAS can be utilized to activate impaired connections.

It is well established that several parameters can influence the excitability of the reflex arc, which poses challenges for repeated H-reflex recordings^37^. As factors like mediating interneurons, pre-synaptic inhibition, and nerve properties can contribute to H-reflex excitability, it is important to keep the subject’s positioning stable and identical and to maintain the same intent, muscle relaxation, and stability as in the previous trial. However, in our study, known limitations were recognized and addressed and the recordings were performed adhering to a strict protocol, which improved the reliability of the H-reflex recordings^30^. For example, the recordings were performed in a standardized relaxed prone position that considered body, head, foot, and other joint angles, using supportive cushioning and reducing the possible error caused by difference in the testing position or unwitting muscle activation^22,38,39^. While the recording electrodes remained intact throughout the entire session, the placement of the manually applied stimulating electrodes was carefully marked on the skin to ensure consistent positioning during all measurement timepoints^40^. Reliability of Hmax measurement was also ensured by increasing the number of stimuli delivered^37,41^.

Kumpulainen et al. (2012) did not observe soleus muscle H-reflex enhancement when soleus-targeted cortical PAS was applied with four different ISIs, which made them conclude that the observed MEP potentiation was of supraspinal origin^42^. Al’joboori et al. (2021) reported a facilitatory effect in the corticospinal excitability when H-reflex was measured before and after repetitive PAS intervention^43^. They applied simultaneous TMS and transcutaneous spinal-cord stimulation, where both stimuli would arrive at the same time to the spinal motoneurons. They concluded that this facilitation was likely due to an increase in spinal excitability. They further speculated that their protocol would be useful in rehabilitative aspects considering individuals with SCI. Dixon et al. (2016) reported altered corticospinal excitability after their transspinal-transcortical and transcortical-transspinal PAS protocols^44^. They presented a potential source for excitability changes in H-reflex being the changed Ia afferent properties and modifications of the excitability profile of the motoneuron pool. Therefore, there are crucial elements in pairing cortical and spinal stimulation and timing intervals. This is consistent with the current study from the perspective of methodology and results.

The maximal M-wave is a compound muscle action potential representing all recruited motor units depolarized by the applied electrical stimulus^45,46^. In the current study, an unexpected finding was observed when Mmax amplitudes increased during the experiment even though the stimulation properties remained unchanged. However, it is important to note that in this study, Hmax was also normalized to its correspondent Mmax, so that the normalization is representative of the currently measured population of motor units allowing the comparison between sessions measured on different days^47^. A significant increase from PRE to POST60 timepoint was found in both sessions in Mmax amplitudes. No significant difference was found between sessions, possibly indicating that the underlying reason was similar in both sessions. This is supported by the correlation analysis, which indicated changes in the Hmax correlating to changes in H/M ratio, whereas changes in Mmax didn’t show similar correlation. As M-wave assesses the peripheral properties and we measured maximal peak-to-peak amplitude in all timepoints, it can be reasoned that the observed effect is of peripheral origin. Rutkove (2000) found that at the baseline temperature, exercise increased median compound muscle action potential^48^. It is possible that environmental factors such as temperature, altered neural drive or synchronization, or factors like changes in architecture of the muscle-tendon complex contributed to the M-wave changes^45,47–49^. In the current study, subjects were instructed to spend the breaks between timepoints similarly. Because of preparation and high-PAS, the preceding time before PRE and POST timepoints were slightly different compared to later timepoints. It is possible that some changes in muscle features may have occurred. As breaks in both sessions followed identical behavioural habits, this might explain the observed effect being homologous and environmental effects remaining sufficiently similar when compared. Therefore, the confounding factor is temperature, which was not controlled or measured from skin, muscle, or surroundings. For future studies, measuring and controlling this factor is suggested.

The spinally targeted high-PAS protocol is designed with the idea of plastic changes occurring at the spinal-cord level between upper and lower motoneurons and their intervening interneurons. In high-PAS, the PNS train is applied with an intensity that is sufficient to activate lower motoneuron cell bodies at the spinal-cord level, which is ensured by F-response measurements^32^. In several of our previous studies involving high-PAS and patients with incomplete SCI, we observed improvements in muscles that are innervated by the stimulated nerve trunk^9,10,17^. Improvements were also found even in muscles that are not directly innervated by the stimulated nerve trunk; thus, activation of the surrounding regions of the motor cortex and activation of cell bodies of neighbouring peripheral nerves is also possible^4,9^. Electrical stimulation is thought to strengthen the connections between neighbouring neurons^5^. In high-PAS, PNS is used at an intensity that is sufficient to elicit F-responses and thus stimulate motoneurons of the spinal cord^24,50^. Therefore, a PNS-induced pulse train applied to a peripheral branch is thought to induce activation of the larger pool of the lower motoneurons and intervening interneurons. Although measurements for high-PAS were made utilizing MEP and F-responses obtained from AH-placed electrodes and stimulation targeting the tibial nerve, our current result of changes in H-reflex pathway measured from soleus confirms the hypothesis that changes occur at the spinal level affecting the targeted myotome. In future studies, antagonist and synergist muscles should be measured for further insight on intermuscular coherence.

Possible overall mechanisms behind the changes in reflex amplitudes are alterations in motoneuron excitability, neurotransmitter release changes, and changes in intrinsic properties of motoneurons^51^. One explanation for the reflex amplitude changes between the SPINAL and the CORTICAL session is that they reflect the changes in the excitability of the motoneuron pool, spinal interneurons (such as weakening of the presynaptic inhibitory interneuron activity), or both, induced in the spinal but not in the cortical session^30,39,52^. Misiaszek (2003) suggests that H-reflex holds promise when acting as a probe to study adaptations with training, injury or disease, or with therapeutic interventions^51^. For example, measuring an informative recruitment curve of H-reflex and M-waves may provide detailed insights into spinal adaptations in future studies. Milder and more recent injuries benefit from shorter high-PAS interventions than older and more severe injuries to gain significant increase in function; however, longer interventions can still lead to increase in function^9,11,53^. While the high-PAS protocol was created with the therapeutic aspect in mind, understanding these underlying mechanisms could help to modify and further develop the protocol to target specific structures in healthy persons and individuals with incomplete SCI.

The small sample size of ten healthy subjects is a limitation of this study. To minimize variability in the results, sessions were scheduled on the same time of day following the subjects’ circadian rhythms to represent a normal day as closely as possible in the subjects’ daily living. Another limitation was the need to change the subject’s position from sitting in MEP assessment to prone in H-reflex assessment. By having another person apply ISI and blinding the H-reflex assessor from session order, potential bias in measurements could have been avoided. Additionally, this study examined the acute effects of the intervention, therefore long-term studies are needed to further understand the effect of high-PAS on spinal-level excitability. The applicability of the results from healthy subjects to individuals with SCI should be tested to determine relevance.

The main purpose of this study was to investigate with H-reflex measurements whether spinally targeted high-PAS could induce acute changes at the spinal level and therefore strengthen the evidence on the assumed mechanisms of the high-PAS protocol that has already led to relevant findings. Additionally, we sought to compare these potential spinal-level changes with those possibly induced by cortically targeted high-PAS. We observed significant increases in Hmax amplitudes during the SPINAL session but not in the CORTICAL session. Our results show that high-PAS is sensitive to the ISI used between the peripheral and cortical stimulation when targeting specific locations. Conventional PAS with high timing dependency is useful in studying corticospinal plasticity in healthy subjects. However, patient populations suffering from spasticity or neuroanatomical differences can influence the timing determination and performance outcome. The results of this study support our previous findings on high-PAS in individuals with incomplete SCI and the overall hypothesis that spinally targeted high-PAS-induced plasticity effects in the somatomotor system are more pronounced at the spinal-cord level than the effects induced by cortically targeted high-PAS. This provides further evidence for high-PAS as an option for clinical settings when targeting plasticity in different levels of the corticospinal tract.

## Electronic supplementary material

Below is the link to the electronic supplementary material.

Supplementary Material 1

## Data Availability

The datasets generated during and/or analysed during the current study are available from the corresponding author on reasonable request.
