# Supplementary material for: Spinally targeted paired associative stimulation with high-frequency peripheral component induces spinal level plasticity in healthy subjects
Source: Sci Rep. 2024 Dec 28;14:31052. doi: 10.1038/s41598-024-82271-4 (PMC11680591; doi:10.1038/s41598-024-82271-4)
Supplement: Supplementary file 1 — Supplementary Material 1 [file 41598_2024_82271_MOESM1_ESM.pdf]

Spinally targeted paired associated stimulation with high-frequency peripheral component induces spinal level plasticity in healthy subjects

Anna Nätkynmäki[1,2], Leena Lauronen[3], Piia Haakana[1,4,5], Erika Kirveskari[1,6], Janne Avela[2,7] and Anastasia Shulga[1,8,*]

*Correspondence: Anastasia Shulga, email: anastasia.shulga@helsinki.fi

Supplementary Table 1.

Table S1. Individual MEP amplitude absolute results from both sessions across all timepoints.

Subject	Session	Timepoint											
		PRE			POST			POST30			POST60		
		AVE	SD	SE	AVE	SD	SE	AVE	SD	SE	AVE	SD	SE
S01	SPINAL	568	269	85	581	216	69	619	249	79	717	472	149
	CORTICAL	552	252	80	789	273	86	450	186	59	950	412	130
S02	SPINAL	1067	325	103	2228	905	286	1545	873	276	1400	546	173
	CORTICAL	1259	173	55	1351	559	177	1319	363	115	1408	378	120
S03	SPINAL	111	92	29	371	272	86	185	176	56	173	110	35
	CORTICAL	135	62	20	267	149	47	259	127	40	329	169	53
S04	SPINAL	414	143	45	441	232	73	647	346	109	450	135	43
	CORTICAL	491	115	36	525	243	77	428	197	62	304	100	35
S05	SPINAL	344	224	77	648	396	125	516	498	158	494	367	116
	CORTICAL	339	208	66	616	534	169	610	286	90	598	540	171
S06	SPINAL	300	158	50	590	309	98	596	254	80	506	275	87
	CORTICAL	317	153	48	319	154	49	378	369	117	398	272	86
S07	SPINAL	301	122	38	702	346	109	224	139	44	216	108	34
	CORTICAL	226	131	42	619	233	74	297	189	60	309	111	35
S08	SPINAL	652	137	100	738	312	99	832	369	117	724	297	94
	CORTICAL	712	238	75	801	248	79	694	215	68	675	245	77
S09	SPINAL	778	510	161	1216	403	127	658	315	100	875	375	118
	CORTICAL	1117	510	161	2174	851	269	1423	739	234	1020	572	181
S010	SPINAL	167	137	43	708	446	141	390	391	124	579	715	226
	CORTICAL	297	199	63	1038	545	172	360	369	117	422	381	121
All	SPINAL	470	296	94	822	543	172	621	382	121	613	352	111
	CORTICAL	541	379	120	850	566	179	622	417	132	641	374	118

Note: AVE, average (µV) representing an average of 30 MEPs; SD, standard deviations; SE, standard error.

Supplementary Figure S1.

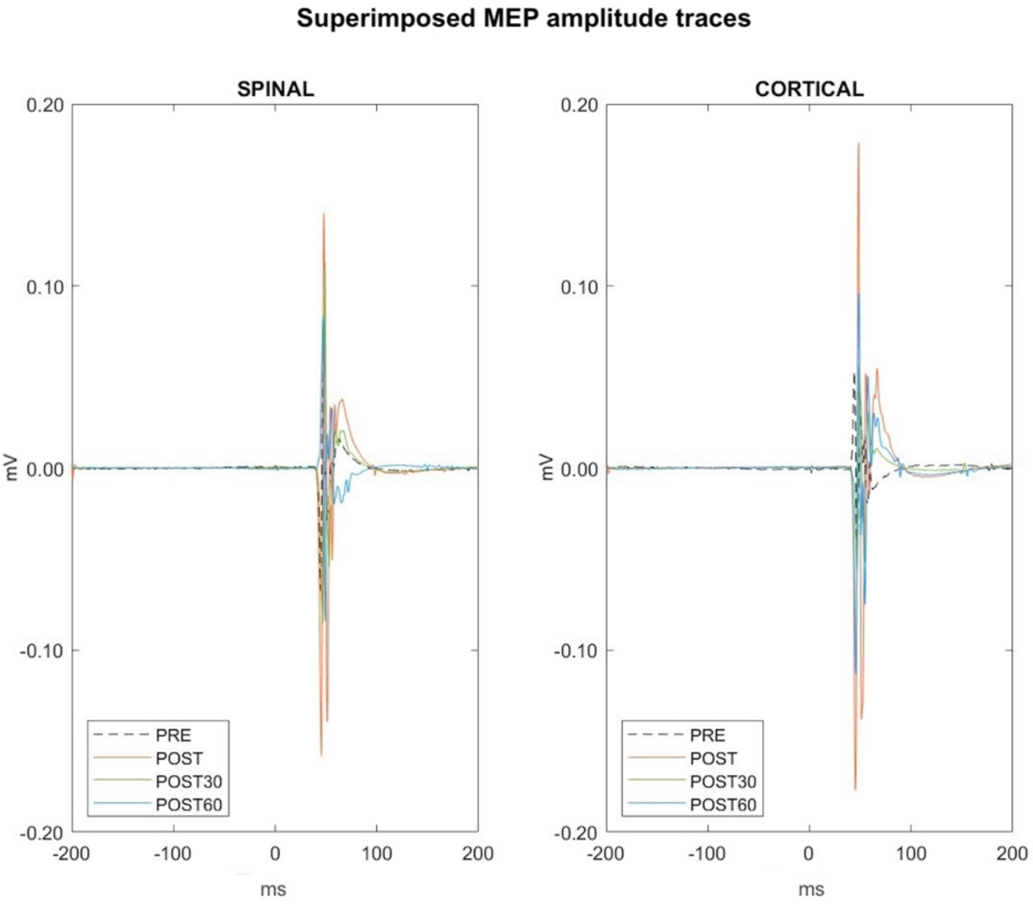

Figure S1. Representative averaged MEP amplitude (mV) traces from SPINAL and CORTICAL session of a pilot subject. The left panel represents the SPINAL session and the right panel the CORTICAL session. Each trace represents an average of 30 MEPs. Detrend function was used to bring prestimulation EMG baseline to 0 mV. 50 Hz noise was filtered with a notch filter. 0 on the x-axis (time) indicates the time of given TMS pulse.

Supplementary Figure S2a.

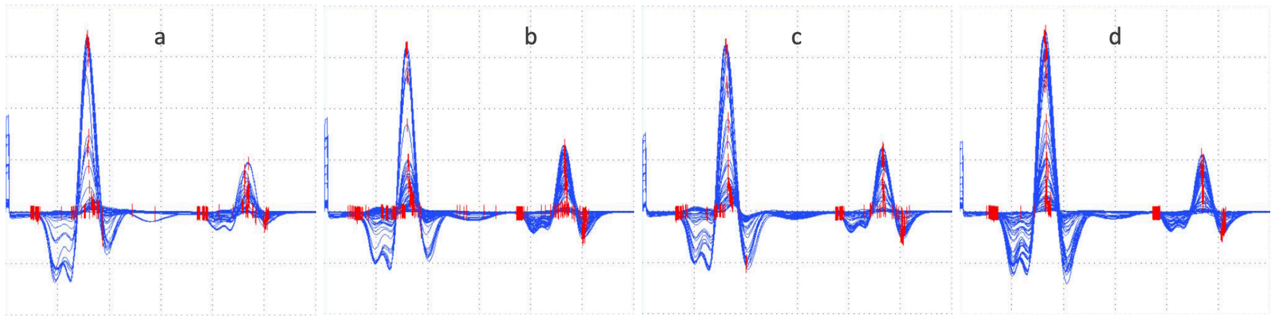

Figure S2a. Representative superimposed (scale 8 ms/D, 2 mv/D) traces showing Mmax and Hmax amplitudes from the SPINAL session at PRE (a), POST (b), POST30 (c), and POST60 (d) timepoint measurements of a pilot subject.

Supplementary Figure S2b.

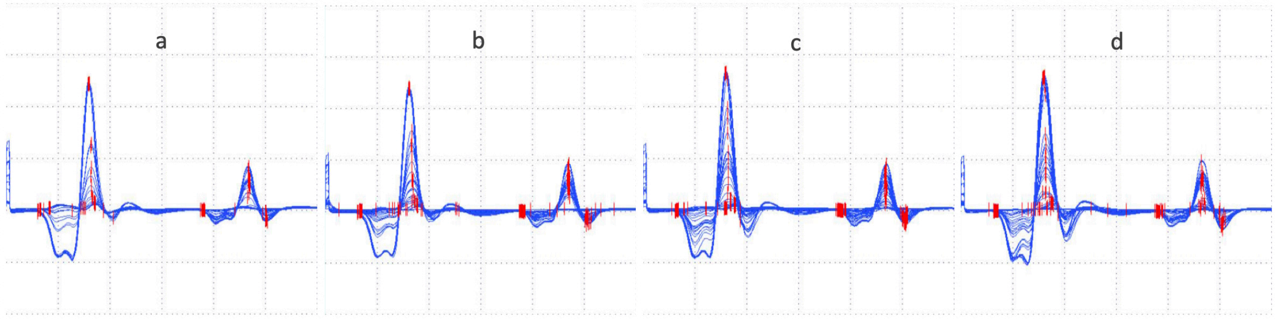

Figure S2b. Representative superimposed (scale 8 ms/D, 2 mv/D) traces showing Mmax and Hmax amplitudes from the CORTICAL session at PRE (a), POST (b), POST30 (c), and POST60 (d) timepoint measurements of a pilot subject.
